# The Role of Partnerships in U.S. Food Policy Council Policy Activities

**DOI:** 10.1371/journal.pone.0122870

**Published:** 2015-04-09

**Authors:** Megan L. Clayton, Shannon Frattaroli, Anne Palmer, Keshia M. Pollack

**Affiliations:** 1 Department of Health, Behavior and Society, The Johns Hopkins Bloomberg School of Public Health, 624 North Broadway Street, Baltimore, Maryland, 21205, United States of America; 2 Department of Health Policy and Management, The Johns Hopkins Bloomberg School of Public Health, 624 North Broadway Street, Baltimore, Maryland, 21205, United States of America; 3 The Johns Hopkins Bloomberg School of Public Health Center for a Livable Future, 615 North Wolfe Street, Suite W7010, Baltimore, Maryland, 21205, United States of America; University of North Carolina at Chapel Hill, UNITED STATES

## Abstract

Food Policy Councils (FPC) help to identify and address the priorities of local, state, and regional food systems with the goal of improving food systems through policy. There is limited research describing FPCs’ strategies for accomplishing this goal. As part of a larger study examining FPC policy efforts, this paper investigates the role of partnerships in food systems policy change. We conducted interviews with representatives from 12 purposefully selected FPCs in the United States and 6 policy experts identified by the selected FPC representatives to document and describe their policy work. One theme that emerged from those interviews was the role of partners. Interviewees described a range of partners (e.g., stakeholders from government, business, and education) and credited FPC partnerships with advancing their policy goals by increasing the visibility and credibility of FPCs, focusing their policy agenda, connecting FPCs to key policy inputs (e.g., local food community knowledge and priorities), and obtaining stakeholder buy-in for policy initiatives. Partnerships were also described as barriers to policy progress when partners were less engaged or had either disproportionate or little influence in a given food sector. Despite these challenges, partnerships were found to be valuable for FPCs efforts to effectively engage in the food policy arena.

## Introduction

Food Policy Councils (FPCs) are an increasingly common and important approach for improving food systems at local, state, and regional levels (i.e., the entity they are targeting with their interventions). In contrast to historical efforts, which managed food issues within related sectors (e.g., production, processing, retail), FPCs work across sectors to address the food system as a whole [1]. This systems-lens recognizes connections among topics that may lead FPCs to focus on policies and programs to address poverty, economic production, hunger, labor, and public health, among others. Cross-sector work also involves people and institutions with different priorities and perceptions regarding food system problems and solutions [2, 3]. FPCs serve a number of interrelated functions, such as providing an opportunity for diverse stakeholder discussion, promoting coordination across sectors, creating new or supporting existing programs and services that attend to local needs, and influencing and evaluating policies [1]. These functions are meant to unite stakeholders under a common food system vision, which is advanced through food policy change and related activities.

Recognizing their growth and increasing presence in food system discussions, some research has sought to understand the needs and challenges of FPC policy engagement [1, 4–6]. According to a 2009 Food First Report, in order to advance food systems policy FPCs must be viewed as credible by decision-makers and earn support from other food system stakeholders. FPCs’ diverse networks, which often include different interests and policy agendas, can be a challenge [1, 6]. Some struggle to navigate thorny political climates; balance policy and program priorities; evaluate their impact; and identify sufficient time, funding, training, and skills to advance their policy agenda [5–8]. FPCs appear to be more effective when they harness local momentum and address needs with local organizations [9]; however, partnering with organizations within the local food environment can take years [1].

The investment in partnerships appears to be worthwhile, as FPCs rely heavily on partners to achieve their missions [6, 7, 9, 10]. FPC partners include individuals, institutions, and agencies that can help to advance FPC priorities. Partners contribute skills, perspectives, and resources to produce knowledge, bolster capacity, and support innovation for addressing complex food systems issues [11, 12]. They may also promote participatory democratic processes by connecting grassroots groups with FPC initiatives [13]. According to a 2012 Canadian study, key food system stakeholders saw partnerships as central to their role in food policymaking [14].

While the literature recognizes the significance of partnerships to FPCs, few studies explore how they influence FPC engagement in the policy process—including challenges and needs associated with partnerships. Here we describe FPC partnerships and document how FPCs describe partners’ roles in the context of food systems policy work.

## Materials and Methods

We conducted a two-part study of FPCs and their policy-related activities. Part 1 included an online survey of U.S. FPCs to describe council policy engagement: the results are published in a separate manuscript [6]. Part 2 was a multiple case study of FPCs identified as engaged in policy work through the online survey. Data collection for the multiple case study involved in-depth interviews with representatives from the selected FPCs, and documents about the selected FPCs available from their websites [6]. This study reports on findings from Part 2.

To describe FPCs, we used the definition provided by Harper et al.: entities that, “serve as forums for discussion of food issues; foster coordination among sectors in the food system; participate in policy processes; and launch or support programs and services that address local needs” [1]. Harper’s definition was chosen based on guidance from content expert advisors on this project.

### Sample

To survey all FPCs in existence as of January 2011 (Part 1), we utilized several sources: a list of FPCs from the Community Food Security Coalition (CFSC); a list of national food policy conference attendees; and the websites of individual FPCs. The CFSC list was the primary source for identifying FPCs and was verified using the food policy conference attendee list and websites of individual FPCs. Additional information on the sampling frame can be found elsewhere [6].

The online survey included a question regarding whether respondents would be willing to participate in an in-depth case study of their FPC. Of the 92 FPCs invited to participate in the survey, 56 completed the survey and 37 (66%) agreed to be contacted for the case study [6]. From these respondents, we purposefully selected a diverse sample based on geographic location (i.e., FPCs representing the West, Midwest, Northeast, and South U.S. Census Bureau regions) and level of policy engagement, including FPCs that described working on a range of policy activities (e.g., developing and lobbying for policy proposals, participating in the regulatory process, and implementing policies) at multiple levels (local—city or county—, state, and national). We also relied on input from two FPC experts who reviewed our preliminary sample to assure it would best inform our understanding of FPCs engagement in policymaking. We used contact information from survey respondents to follow-up with select FPC representatives by email. Our sampling approach was non-representative and purposively developed to include participants most able to provide rich information to inform the research questions.

We identified 12 FPCs that reflected equal representation of different levels of work (local, state, and regional) and that were prioritized within each scope of activity category by length of existence; brand new FPCs were not included. We sent each FPC representative an email invitation to participate in the study as a key informant interviewee. All FPCs contacted agreed to participate with the exception of one FPC at the local level, which we did not hear from despite multiple attempts at contact. All FPC contacts were in a leadership position within their councils. Based on recommendations from FPC representatives, we included policy experts, defined as persons with authoritative knowledge and experience in the policymaking process, who worked closely with 5 of the participating FPCs (both as FPC members and non-members) to advance legislative or regulatory policies consistent with the FPC mission. We invited these candidate interviewees to participate in a key informant interview. Of the 5 interviewees who identified policy experts for our sample, 4 referred us to1 expert and 1 identified 2 experts. All 6 policy experts agreed to participate.

### Data Collection

All four co-authors conducted the interviews with at least two authors participating in each interview. For interviews with FPC representatives, we created an interview guide based on survey findings and the overall study aims. The interview guide included general questions about the FPC’s history and organizational structure, how and why FPCs engage in specific policies, barriers and opportunities to policy engagement, development of and strategy for advancing FPC priorities through partnerships, and advice regarding effective engagement in food systems policy.

Given that policy experts may provide greater depth of information on the policymaking process, we modified the policy expert interview guide to focus on questions regarding policy engagement. The interview questions for FPC and policy expert participants are listed in Table 1. Interviews occurred in person at the May 2011 Community Food Security Coalition meeting and via telephone from August 2011 to December 2011. One additional in-person interview with a local FPC was conducted in November 2011. Policy expert interviews occurred via telephone from October 2012 to December 2012. Interviews were recorded and transcribed.

**Table 1:** Interview Guide Questions for FPC and Policy Expert Participants.

FPC Interview Questions	
Introductory Questions	*Verify from Part 1 Survey*: *FPC name; time in existence*
	*How did the FPC start*?
	*What is your role in the FPC*?
	*Do you have staff*?
	*What type of organization is your FPC*?
	*Tell me about the membership of your FPC*.
	*What is the role of the community in the FPC*?
Activities of the Food Policy Council	*Besides policy work*, *what kind of work does the FPC do*?
	*How does the FPC identify priorities for action*?
Policy Work of the Food Policy Council	*How would you describe your policy objectives*?
	*If you have funds to support your FPC*, *roughly what percentage of FPC funding is spent advancing policy*?
	*How would you describe your FPC’s process of engaging in the policy process*?
	*What policy initiatives are currently underway within your FPC*?
	*Thinking about the list of current policy initiatives*, *what are the 3 most important policies initiatives and why*?
	*What role does your FPC have in policy implementation or assuring that policies are translated from words to action*?
Reflections on Policy Initiative/Lessons Learned	*Based on your experience*, *what are the key factors for success in the policy arena for your FPC*?
	*What are the challenges your FPC encounters when working to advance policy*?
	*What advice would you give to an FPC interested in beginning to work on policy issues*?
	*Are there gaps between what you (or the FPC founders) hoped for the FPC and the reality of the FPC work at this point*?
Conclusions	*Are there written materials available that would help me to better understand the FPC’s policy work*?
	*Is there anything else you would like to tell me about your FPC and the policy work you do*?
	*Is there anyone else I should talk with in order to understand your FPC and the policy work you do*?
Policy Expert Interview Questions	
Introductory Questions	*Can you describe when and how you were first introduced to the food policy council work*?
	*Can you describe your role on the food policy council*?
	*Why does this work matter to you*?
General Thoughts on FPC Policy	*What is your biggest achievement on the council*?
	*What is your biggest policy achievement*?
	*What policies have you found particularly challenging*?
Specific Policy Initiative(s)	*When we interviewed [FPC representative]*, *he/she told us about the work you did together on [specific policy initiative]*. *Can you describe that initiative*? *Why did you get involved in this specific initiative*?
Conclusions	*What advice would you give to an FPC Director about how to engage with policymakers (or decision-maker if interviewee is not a policymaker)*?
	*If you were talking to a group of legislators*, *would you tell them to include food policy in their agenda*? *Why or why not*?
	*Is there anything else you would like to tell us about your work*?

### Ethics Statement

The Johns Hopkins Bloomberg School of Public Health Institutional Review Board reviewed and approved the study and procedures, and determined the research to be "Not Human Subjects Research." The research involved questions regarding people’s professional knowledge and experiences and did not involve the collection of any personal data. We included the oral consent process as a means of explaining the research and how we would use the information provided, but due to the minimal risk associated with the content of the work, opted not to use a written consent procedure. We also determined that the oral consent process was more conducive to the conversational style of the interviews that we sought to achieve. All participants agreed to the oral consent, as we would not have proceeded without their approval, which included approval to audio record the interview.

### Coding and Analysis

Transcripts were validated to assure accuracy. One co-author reviewed the transcripts and developed an initial coding dictionary. The four co-authors reviewed an initial subset of transcripts for the purpose of assessing the completeness of the coding dictionary and refining the associated definitions. Using an iterative process, we met regularly to compare our application of the codes, discuss inconsistencies, and revise the codes and definitions until we were applying the codes consistently. Using this revised dictionary, we formed two teams of two coders, divided the transcripts between the two groups, and double-coded each transcript. We compared the codes within teams for each subset of transcripts.

With the coding complete, we divided the codes among the four co-authors who each reviewed the coded text associated with their assigned codes. The purpose of this stage of the analysis was to analyze the coded text across transcripts and obtain a summary understanding of the coded data. The co-authors then met to discuss their analysis and interpretation of their assigned codes. Together, using a content analysis approach, the team identified common themes and exemplary quotes, and developed a list of aims for analyses. Coding and analysis were organized using ATLAS.ti qualitative data analysis and research software [15].

## Results

Our final FPC sample consisted of 12 FPCs organized at the regional (n = 4), state (n = 4), and local levels (n = 4) from the West (n = 3), Northeast (n = 2), Midwest (n = 5), and South (n = 2) U.S. Census Bureau regions (Table 2). All FPCs reported some level of engagement in policy activities. Some were affiliated directly with government (n = 4), others were non-governmental organizations, including those created and authorized by a government entity operating independent of government (n = 8). Most (n = 10) had been in existence for less than ten years (2–5 years: n = 6; 6–10 years: n = 4; 10+ years: n = 2).

**Table 2:** Characteristics of FPCs Interviewed**.

Characteristic	No. (%) or Mean (Range)
No. Years in Existence	8.13 years (2–30)
Level Organized	
Regional	4 (33.3)
State	4 (33.3)
City/local	4 (33.3)
Geographic Distribution	
West	3 (25.0)
Northeast	2 (16.7)
Midwest	5 (41.6)
South	2 (16.7)
Reported Level of Policy Activities*	
Federal	3 (25.0)
Regional	1 (8.3)
State	5 (41.6)
Local	10 (83.3)
Organizational Affiliation	
Government	4 (33.3)
Independent	8 (66.7)

*Numbers do not equal 12 because FPCs reported engaging at multiple levels of policy (i.e., local—city or county, state, or regional)

** Current as of 2013

Our policy expert sample consisted of 6 people identified by 5 FPCs located in the West (n = 2), Midwest (n = 2), and South (n = 1) U.S. Census Bureau regions. Five policy experts worked in government positions at the federal (n = 2) or local (n = 3) levels; the sixth worked in the private sector.

Study results are organized into two main sections. First, we outline the types of partners and conceptualizations of partnership as defined by FPC representatives. These results help to contextualize the identified themes regarding FPCs’ perceptions of the role of partnerships in policy engagement. These themes, which comprise section two of our results, are organized according to four thematic categories: (1) partnerships as an opportunity for visibility and legitimacy in policy work, (2) partnerships as providing focus for the policy agenda, (3) partnerships as access to important policy inputs, and (4) partnerships as a facilitator of stakeholder buy-in.

### Types of Partners

FPC representatives identified partners in a variety of sectors and disciplines. They also spoke broadly about partnership with the community. Though they did not always define who they saw as their community, they described community partners as local organizations, council members, and residents or, “the folks growing the food on the vacant lots.” Only a few FPCs discussed partners in other FPCs, though when mentioned, these connections were seen as important to link councils and acknowledged as “communities that are doing similar work.” Table 3 describes the types of individuals or groups mentioned as FPC partners.

**Table 3:** FPC Partnership Types.

Sector	Level or Category	Partners
Government	Federal	USDA Rural Development Offices, Regional Directors
	State	Department of Agriculture, Corrections, Education, Community Health, Health and Social Services, Environmental Quality, Public Assistance, Division of Economic Development, State Legislators
	Local	Board of Alderman, City & County Council, City Law Department, County Commissioners, Parks Department, Planning Commission, Public Health Department, Public Service Department, Office of Neighborhoods, Office of Sustainability, Office of Transportation
Education	State and Private Universities, Extension Programs	Cooperative Extension, Departments or Institutes of Community-Based Participatory Research, Food Systems Research, Law, Medicine, Obesity Research, Planning, and Public Health
	Local School Systems	High School Directors of School Nutrition Services and Science Curriculums
Commercially-Oriented Organizations	Local	Chamber of Commerce
Civic-Oriented and Other Organizations	State	Center for The Environment
	Local	Corporate Accountability Groups, County Land Banks, Farmers’ Markets, Food Pantries, Micro-business Centers for Food-Related Entrepreneurs, Soup Kitchens
Other (Cross-Sector)	State	FPCs
	Local	FPCs, Local Residents Who Grow Food

### FPC Descriptions of Partnership in Relation to Policy Work

FPC interviewees talked about partnerships in a variety of ways. Four main themes emerged to capture these relationships as they related to FPC policy engagement.

#### Participating in FPC meetings

Most respondents talked about partners as individuals or groups who regularly attended council meetings or who only participated in specific strategic meetings to consider policy priorities. These partners as FPC members most often included people from local government, the school system, community groups, and businesses. More strategically-engaged partners were often representatives from state and federal government.

#### Advising and connecting stakeholder groups

Representatives also described partners as individuals or groups who engaged with FPCs through external activities. For example, some FPC representatives talked about partnership with a local agency through serving on a special committee, such as a food task force, or through interacting with community and school groups to provide programmatic or grant-writing support. A number of FPC interviewees also described partnerships that connected groups across sectors to help them identify policy opportunities. According to one participant from a state-level FPC located in the West, these partnerships advanced FPC policy goals by linking others’ “on the ground practical work” to “the policy arena.”

#### Connecting with key policy stakeholders

Other descriptions of partnership related specifically to people with the ability to directly and immediately impact FPC policy goals, such as policy experts. FPC respondents described productive partnerships with policy experts through success in collaboratively writing a resolution or a bill, or through introducing FPC representatives to policymakers with the authority to change current agency practices.

#### Strategic FPC representation in the policymaking arena

A couple of state and regional-level FPCs defined policy-relevant partnerships in terms of who the partner represents. For one interviewee it was imperative that FPC partners include the community or policy solutions would be “bound for failure.” This intentional inclusion of particular partners was echoed by one state-level respondent who defined “true partnership” as relationships with diverse stakeholders from across the food system (i.e. farmers, retailers, consumers), including those who understand government structure and processes. As this interviewee explained,

*When it comes to building food policy councils, partnership is intentional. If it's all private sector then I consider it more special interest. If it's both public and private it means that we're learning about how government works and how different institutions within government work, what the challenges are, what the benefits are and how through legislation of policy we can actually improve or change things that are really about laws or statutes or codes.*

For several FPCs representatives interviewed, partnership was strategic and deliberate, and clearly intended to increase FPC influence in policy decisions.

### Partnerships as Opportunity for Visibility and Legitimacy in Policy Work

Some FPC respondents described partnerships with government leaders and researchers as important for achieving greater visibility and legitimacy for FPC policy efforts. We interviewed these 6 policy expert partners to complement FPC representatives’ perspectives and to provide another perspective on the function and influence of these relationships.

#### Entry to food policy arena through policy expert partners

One federal agency representative described how partnering with government representatives can raise awareness among policymakers about FPCs and their potential contribution to policy discussions. Partners’ access to ‘higher level’ stakeholders (e.g., state legislators and agency leaders) and their familiarity with policy tools (e.g., statements of support) can create new opportunities for FPCs. As this policy expert explained,

*…because of my leadership position and my political position I have access to higher levels. For example, when we had our first meetings on the food policy council we were looking for a way to demonstrate that this is a good thing. A lot of people in [the state] didn't know what a food policy council was. We felt like we were just introducing the concept to the public or to policymakers, state legislators. I didn't know what a food policy council was so you look for a way to sort of have second verification. Anyway, I contacted our Secretary's office and I got a written statement.*

In cases where FPCs don’t have policymakers as partners, they may still build reputations as food policy stakeholders through partnership in government-related groups. As one local-level FPC interviewee explained,

*A lot of what we’re working on, in terms of overcoming challenges, is related to […] gaining legitimacy in the eyes of our policymakers and local institutions. So I see the fact that you know, we are participating again on the kind of internal technical advisory group for the city, around urban ag, as a win on that front.*

These interviewees describe the flexibility of partnerships. For example, for advancing FPC status in the food policy arena, interviewees described partnerships when referring to connecting with key policy stakeholders or advising stakeholder groups.

#### Strategic policy engagement

Both our policy expert and FPC interviewees expressed how partnerships with government leaders and researchers may promote FPC legitimacy through strategic policy engagement. One policy expert suggested that he could identify when policymakers are ready to initiate a new policy idea and help FPCs take advantage of that opportunity by increasing awareness about a problem and a potential solution. Partners can also steer FPCs away from controversial issues, which may preserve FPCs’ legitimacy in the policy arena, as one representative from a local-level FPC located in the Midwest explained,

*[This policymaker has] been really great and really protective to make sure that the food policy council doesn’t become the advocacy arm of that policy and then be forever identified with this really contentious [issue].*

Finally, a representative from a local-level FPC discussed how effective engagement in the food policy arena meant sharing clear, evidence-based recommendations with policymakers. Research partners were seen as essential for effective FPC-policymaker communication that involves interpreting the available evidence.

### Partnerships as Providing Focus for the Policy Agenda

Some respondents, including FPCs with strong policy expert partnerships, attributed pressure to develop a focused policy agenda to specific partner types, including policy experts and national organizations. This process was described through two main mechanisms: weighing in on the policy agenda and focusing FPCs on their policy end goal.

#### Informing the policy agenda

A few FPCs talked about the role of policy expert partners in helping them to prioritize their policy agenda. For one representative from a local-level, Midwest-based FPC with a policy partner, this meant engaging partners in FPC meetings or other activities. For another local-level, Southern FPC without a policy partner, they obtained input on their policy agenda by sending the agenda to “all the elected officials in [the] community” and asking for input.

While policy partners are comfortable helping FPCs prioritize their policy goals, their involvement is limited when it comes to operationalizing the identified priorities. As one policy expert explained,

*So none of the commissioners, including myself, are really hands-on at this point; it really is up to [the FPC] to keep moving forward, but any one of us can suggest projects or policies that we’d like to push on…*

With policy priorities identified, the FPCs’ work involves activities to inform policy decisions, sometimes alongside advocacy groups. Policy partners within government may have a conflict with their own organizational (in the case of agency representatives) or professional (for legislators) responsibilities. Thus, recognizing the boundaries of an acceptable partner role for government officials is important. They should be informed about issues that stakeholder groups such as FPCs view as important and engaged in dialogue without being overly influenced or driven by them. The need to divide responsibilities at different stages of the policy process is demonstrated through this observation that different partners are able to fill different roles, and is one concrete example of the value of partnerships in FPCs’ policy efforts.

#### Focusing councils on policy end goal

A few FPC and policy expert respondents talked about the potential for FPCs to get distracted by the process and lose site of the end goal when FPCs have had “a little bit of time on the ground” and become involved in issue politics. These respondents, all associated with state-level FPCs and engaged in state and federal-level policy work, felt partnerships with policy experts and national organizations helped to refocus FPCs on a policy end goal. As one policy expert explained,

*Policy is a much bigger, bigger lever. So I think I kind of bring that perspective too, we’re doing all this, we’re bringing these stakeholders together to look at our food system but for the end goal of making policy change and encouraging our policymakers to do that.*

Policy expert partners may be important catalysts for moving FPCs beyond meetings and programmatic activities to engaging the FPC with policymakers and the policy process.

### Partnerships as Access to Important Policy Inputs

FPC representatives from all U.S. regions discussed the opportunities and challenges of accessing various inputs to policy work through different types of partners. Interviewees described these inputs within two main categories: building the right policy infrastructure and revealing stakeholder knowledge, needs, and interests.

### Building Policy Infrastructure

One representative from a local-level FPC located in the West that did not have policy expert partners, talked about the need to have “the right infrastructure in place” to do policy work. According to this interviewee, strategic partners who can give FPCs access to stakeholders, provide strategic policy support, and increase an FPC’s capacity to engage in time consuming policy processes are an essential part of the infrastructure that FPCs do not have on their own.

*If we're interested in working on school food policy, either [the director of nutrition services for the school district] needs to be on the council, or we need to have good relationships with [them]. And kind of identifying, not just the issue areas that you want to work with, but the people who you want to work with is really, really important. I mean, this is very much driven by being able to kind of, again, rely on partners and work through networks, and access power.*

Another representative from a local-level Midwestern FPC further detailed this point through her description of the specific influences each partner brought to the table in passing a new zoning code. Building the right policy infrastructure required seven different types of partners (e.g., local residents including community gardeners, the public health department, director of planning, office of sustainability, local council members, academics, and topical experts) who engaged in a range of tasks (e.g., writing the zoning code, providing input on enforcement, researching, educating, and answering questions, testifying, lending strategic support) over an extended period of time.

According to some FPC representatives, however, the process of finding partners who may help build the right policy infrastructure is not always straightforward. One interviewee from a local-level FPC described two major, interrelated factors that complicate access to resources: (1) partners’ commitment to the FPC mission; and (2) the FPC’s ability to diversify their partners to include key policy experts. Based on her experience engaging in food system policy in the Northeast, one interviewee explained,

*We haven't gone to them with an official bill yet, so this is going to be our first foray into that. We have always had a liaison from the Board of Alderman to the food policy council. The alderman who's involved with us right now is particularly interested in these issues, and so it's been very active. We—our past alder people have not—they've been very interested, but they just haven't had time to work with us. And so what we're hoping to do in the future, and again it all depends on how much time and energy people have to do this, is begin to build relationships with more of the alder people rather than just the one liaison, because right now we're counting on him to help us spread the word amongst the rest of the alders.*

Another regional-level FPC expanded on the potential negative consequences of these complicating factors. In partnering with the local chamber of commerce, the interviewee described how this partner limited their local-level FPC’s access to the business community by claiming to “speak as the business community voice, whether they're really doing it or not.” This idea that partnering with an organization implies that the organization is the FPC’s gateway to related members created a barrier to accessing other partners in that community. Another interviewee from a state-level FPC suggested that challenging power dynamics are not uncommon, since, “You’ve got certain pieces of the food sector that are bigger and stronger and more politically viable and have been around longer.” For this interviewee, such imbalances in stakeholder influence challenge the FPC’s ability to get partners to respect and accept one another.

In the case of inter-FPC relationships, the motivation to achieve policy-relevant power through partnership may motivate certain FPCs to avoid relationships with less active counterparts. For example, a representative from a regional-level FPC that had worked on local-level policies praised a new collaboration with an FPC that was bigger, better organized, and had a skilled policymaker at the helm. When asked about partnering with another local FPC that was less active, prioritized networking over advocacy, and had limited policy experience, this interviewee explained, “I don’t think they have any power right now,” suggesting such an alliance would not be in the interviewee’s best interest.

#### Revealing stakeholder knowledge, needs, and interests

Several representatives from local-level FPCs found that partnerships were important for aligning FPC’s policy goals with stakeholders’ knowledge, needs, and interests; the nature of this influence differed based on partner type. For example, a representative from a Midwestern FPC talked about partnering with a non-profit group that inventories vacant land, and credited this group’s land system with informing the FPC’s approach to urban agriculture policy. For others, relationships with the community uncovered locally-relevant targets for policy change. One representative from a Southern FPC explained,

*[Local urban gardeners] identified lots of different policy barriers, zoning, land acquisition, things like the water, and utility company policies around community gardens. There were whole lists of different policy issues they identified.*

In other cases, partners from the private sector provided a market-based context for FPC policy initiatives. In discussing school food procurement policies, one FPC interviewee explained,

*…somebody says, “Well, the school lunches are unhealthy” and [the representative from the school district] is like, “I plan those and I have $1.10 to work with so give me a break here.” So there's that tension, I think, a little bit of the public sector employees having to play reality check on that. Then the business community brings up the very real point, which is, "Yeah, I would love to sell local food. I would love to sell to the school district but, for $1.10, I don't want that business.”*

Through partnerships with a range of stakeholders, including representatives from public and private sectors, FPCs may more effectively develop policies that consider the realities of stakeholders in the local food community.

### Partnership as a Facilitator of Stakeholder Buy-In

Many FPC interviewees, at both local and state levels, described partnership as important for stakeholder policy and programmatic engagement. Stakeholders were more likely to be collaborative when engagement was framed as ‘partnership’ and the goal was a shared policy agenda. One local-level FPC interviewee explained,

*And, I think, it allowed a better relationship to be built with the staff particularly [in local government] because they realized that we saw them as partners in helping transformation happen, but that we were not threatened or accusatory.*

According to this respondent, the partnership label may be especially salient to partners from government who may see the FPC initiatives and policy engagement as an indictment of a lack of effort or effectiveness of government systems.

In addition to helping focus policy agendas, some FPC respondents felt the process of pulling partners into the folds of FPC work helped to build commitment to the FPC mission. For example, one representative from a Midwest FPC explained,

*It’s been really great to invite [county administrators] to our meetings. I think that’s helped them have buy-in, because they got to say, ‘we should work on this, stop working on this.’*

Many FPC respondents qualified their statements by remarking that the process of developing partnerships takes time. As advised by one respondent from a state-level FPC that works on policy at all levels and has strong policy expert partners, when FPCs are forming, “It takes time to help people understand it, get involved in the community, [and] get that kind of commitment from the community that this is an important thing to do.”

## Discussion

The growth of FPCs and food system policies is an issue of national interest. As part of a larger study examining U.S. FPC policy initiatives, the present analysis explored the role of partnerships in FPC policy work. In contrast to existing research on FPC partnerships, this is the first study to explore FPC representatives’ perceptions regarding how these relationships may influence policy engagement. Despite some challenges, participants described partners as both essential and beneficial to advancing their food policy mission.

Our results suggest that the FPC representatives we interviewed engage in partnership with a variety of food systems stakeholders, including government officials (legislators and regulators), educators, and community organizations. All FPCs reported partnering with public sector organizations, though the majority of these relationships were with officials at the local-level; only a few FPC interviewees had state or federal-level government partners. FPC representatives described partnership as they understood it, and our results show a wide range of partnerships that are relevant to policy work.

The identified benefits of partnerships may help FPCs overcome some of the documented barriers to policy engagement [1, 4–6]. Previous research supports the notion that partnerships with high-level leaders (such as policymakers and researchers) add credibility to the FPC mission [1, 16]. This study furthers that understanding by clarifying ways these relationships may increase policymaker awareness and FPC legitimacy—specifically with regard to engaging in food systems policy advocacy.

Our findings also suggest that partnerships with policy experts may help FPCs to respond to the unique needs of local policymakers by identifying areas in need of a policy response and to avoid contentious policy issues by serving as a barometer of public opinion on policy proposals. These opportunities for strategic engagement help FPCs tailor their policy efforts to the needs and interests of the local political context and may increase FPCs’ visibility and legitimacy as a key stakeholder in the food policy arena. In partnership with the community, interviewees described a critical opportunity to connect FPC policy priorities with the needs and interests of organizations within the local food environment—a benefit that some interviewees attributed to the difference between success and failure in food systems policy change [1, 9].

Though previous studies found FPCs’ diverse networks to be a challenge to policy engagement, participants in our study suggested that partnerships may advance policy goals by focusing and prioritizing the policy agenda, keeping FPCs working toward a policy end goal, and as a label or frame signaling teamwork for improved collaboration [1, 6]. As our interviews did not capture the content of finalized policy agendas, it is unclear how this process may have influenced representation of diverse stakeholder priorities and interests in FPC programs and policy initiatives.

These findings seemed to vary by partner type and partnership definition, suggesting that partner composition is important for accessing partner benefits for policy engagement. For example, while a range of partners seemed to be relevant for informing and prioritizing a food policy agenda, only a few partner types—such as policy experts and researchers—were seen as critical to building legitimacy and visibility in the policy arena and for engaging with legislative bodies. Though only a couple of FPC representatives talked about relationships with other FPCs, access to a skilled policymaker seemed to signal value in these collaborations. Thus, prioritizing development of these kinds of partnerships may be important policy strategies for newer FPCs and those seeking greater involvement in policy processes.

Overall, the varied ways that partnership across partner types may help address barriers and create opportunities to advance policy engagement represent important points of consideration for evaluating FPC’s effectiveness in achieving their goals. Further, given that our study focuses on partnerships already in place, and finds that their influence on policy engagement may differ by partner type, FPCs may benefit from future research that explores strategies for identifying, engaging, and maintaining partnerships with various partner types, such as policy experts and the community.

This study has some limitations. Our findings are based on a small sample of FPCs, though the selection was intentionally diverse and allowed for in-depth examination of FPCs engaged in policy activities. The FPCs that volunteered to participate were similar to those that did not, except that the ones that volunteered had been in existence for at least a year. It is not surprising that newer FPCs did not self-select for the case study; it is likely that the individuals completing the survey did not yet feel they had enough policy experience to participate in research about FPC policy activities. Because our sample included 12 FPCs, the lessons drawn from the data speak to general processes involving partnership and policymaking, and not to specific scenarios or policies. As such, the findings can be used to inform theories of FPC policymaking, and should not be interpreted as lessons that are generalizable to all FPCs, or FPCs pursuing particular policy goals. Further, we did not use a standard definition of partnership, though this approach allowed interviewees to describe how they operationalize the concept, especially regarding their policy activities.

## Conclusion

This study fills an important gap in the literature about U.S. FPCs by exploring how partnerships influence and advance FPC policy goals. This research also contributes to the analytic generalization of partnerships in the context of food systems work. Our findings may support new and existing FPCs in navigating partnerships to achieve their policy missions. These data may also be of value to other members of the community, research institutions, consumer advocate groups, and local, state, and federal governments that address food systems issues and promote food policy change.
